# Empagliflozin for the treatment of non-alcoholic fatty liver disease: a meta-analysis of randomized controlled trials

**DOI:** 10.4314/ahs.v22i3.42

**Published:** 2022-09

**Authors:** Xue Tang, Huaping Zhang, Xin Wang, Dan Yang

**Affiliations:** 1 Department of Gastroenterology, Qianjiang Central Hospital of ChongQing,400900, China; 2 Department of Obstetrics, Qianjiang Central Hospital of ChongQing; 3 Department of General Medicine, Qianjiang Central Hospital of ChongQing,400900, China

**Keywords:** empagliflozin, non-alcoholic fatty liver disease, randomized controlled trials, meta-analysis

## Abstract

The efficacy of empagliflozin for non-alcoholic fatty liver disease remains controversial. This meta-analysis aims to explore the influence of empagliflozin versus placebo on the treatment of non-alcoholic fatty liver disease and we have searched PubMed, EMbase, Web of science, EBSCO, and Cochrane library databases through July 2021 for related randomized controlled trials (RCTs). Three RCTs involving 212 patients are included in the meta-analysis. Compared with control group for non-alcoholic fatty liver disease, empagliflozin treatment has no improvement in controlled attenuation parameter (CAP) score, hepatic steatosis and liver stiffness measurement (LSM) score, alanine aminotransferase (ALT), aspartate-aminotransferase (AST), low density lipoprotein (LDL) or triglyceride (TG). These indicate that empagliflozin treatment may be not effective for non-alcoholic fatty liver disease.

## Introduction

Similar to obesity, the prevalence of non-alcoholic fatty liver disease has been increasing worldwide for the past 30 years^1^–^3^. Nonalcoholic steatohepatitis is the progressive form of nonalcoholic fatty liver disease and has the features of hepatocellular damage, inflammation, and liver fibrosis^4^–^6^. The prevalence of ultrasound-determined fatty liver in patients with type 2 diabetes mellitus is estimated to range from 29.6 to 87.1%^7^. Serious non-alcoholic fatty liver disease can progress to cirrhosis, end-stage liver disease and hepatocellular carcinoma. Liver diseases are anticipated to become the main cause of mortality in the next 20 years and an important cause for liver transplantation in the next few years^8^, ^9^. Recent findings suggest that non-alcoholic fatty liver disease is a major cause of cryptogenic cirrhosis^10^.

Sodium-glucose co-transporter 2 (SGLT2) inhibitors are reported to increase urinary glucose excretion and decrease blood glucose and insulin levels^11^, ^12^. They result in a significant increase in fatty acid (FA) mobilization from adipose tissues and FA uptake^13^. As one important SGLT2 inhibitor, the beneficial effects of empagliflozin on liver are seen in patients with non-alcoholic fatty liver disease^14^, ^15^.

However, the benefit of empagliflozin for nonalcoholic fatty liver disease has not been well established and several studies reports the conflicting results^15^–^17^. With accumulating evidence, we therefore perform a meta-analysis of RCTs to explore the efficacy of empagliflozin versus placebo for nonalcoholic fatty liver disease.

## Materials and methods

Ethical approval and patient consent are not required because this is a meta-analysis of previously published studies. The meta-analysis are conducted according to PRISMA (Preferred Reporting Items for Systematic Reviews and Meta-Analyses)^18^.

### Search strategy and study selection

Two investigators have independently searched the following databases (inception to July 2021): Pub Med, EMbase, Web of science, EBSCO and Cochrane library databases. The electronic search strategy is conducted using the following keywords: “liver disease” OR “steatohepatitis” AND “empagliflozin”. We also check the reference lists of the screened full-text studies to identify other potentially eligible trials.

The inclusive selection criteria are as follows: (i) patients are diagnosed with non-alcoholic fatty liver disease; (ii) intervention treatments are empagliflozin versus placebo; (iii) study design is RCT.

### Data extraction and outcome measures

We have extracted the following information: author, number of patients, age, female, body mass index, statin use and detail methods in each group etc. Data have been extracted independently by two investigators, and discrepancies are resolved by consensus. The primary outcomes are controlled attenuation parameter (CAP) score to evaluate hepatic steatosis and liver stiffness measurement (LSM) to assess fibrosis. Secondary outcomes include alanine aminotransferase (ALT), aspartate-aminotransferase (AST), low density lipoprotein (LDL) and triglyceride (TG).

### Quality assessment in individual studies

Methodological quality of the included studies is independently evaluated using the modified Jadad scale^19^. There are 3 items for Jadad scale: randomization (0–2 points), blinding (0–2 points), dropouts and withdrawals (0–1 points). The score of Jadad Scale varies from 0 to 5 points. An article with Jadad score≤2 is considered to have low quality. If the Jadad score≥3, the study is thought to have high quality^20^.

### Statistical analysis

We estimate the standard mean difference (SMD) with 95% confidence interval (CI) for all continuous outcomes. The random-effects model is used regardless of heterogeneity. Heterogeneity is reported using the I2 statistic, and I2 > 50% indicates significant heterogeneity^21^. Whenever significant heterogeneity is present, we search for potential sources of heterogeneity via omitting one study in turn for the meta-analysis or performing subgroup analysis. All statistical analyses are performed using Review Manager Version 5.3 (The Cochrane Collaboration, Software Update, Oxford, UK).

## Results

### Literature search, study characteristics and quality assessment

**Figure. 1:**
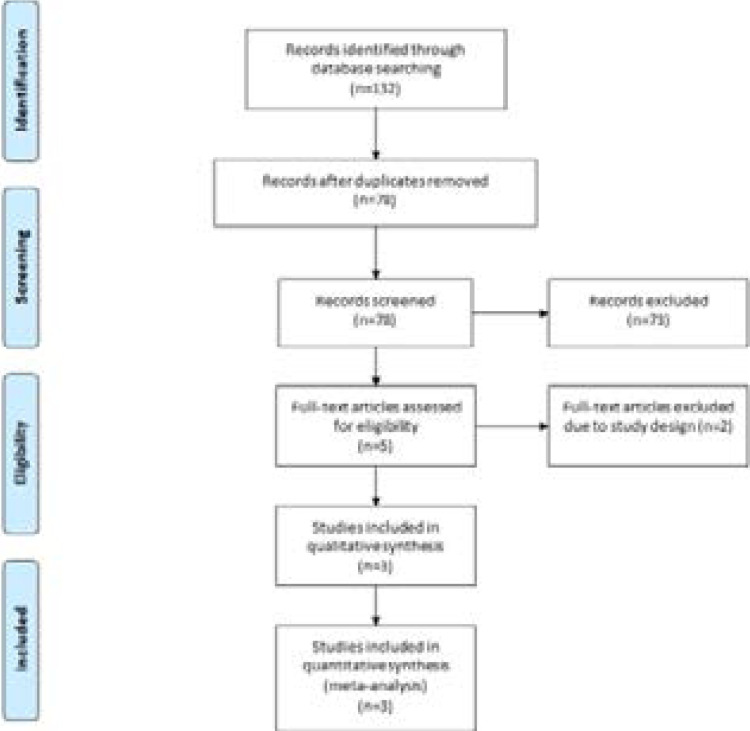
Flow diagram of study searching and selection process.

A detailed flowchart of the search and selection results is shown in Figure 1. 132 potentially relevant articles are identified initially. Finally, three RCTs are included in the meta-analysis^15^–^17^.

The baseline characteristics of three eligible RCTs in the meta-analysis are summarized in Table 1. The three studies are published between 2018 and 2021, and sample sizes range from 50 to 90 with a total of 212. Two RCTs report all included patients with diabetes^15^, ^16^, while the remaining RCT report patients without diabetes^17^. Empagliflozin is administered at the dose of 10 mg daily.

**Table 1:** Characteristics of included studies

NO.	Author	Empagliflozin group						Control group						Jada scores
		No	Age (years)	Female (n)	Body mass index (kg/m^2^)	Statin use (n)	Methods	No	Age (years)	Female (n)	Body mass index (kg/m^2^)	Statin use (n)	Methods	
1	Chehrehgosha 2021	35	50.5±8.4	20	30.9±3.3	34	empagliflozin 10 mg daily for 24 weeks	37	51.8±7.8	23	30.2±4.4	35	placebo	5
2	Taheri 2020	43	43.8±9.7	15	30.5±2.3	5	empagliflozin (10 mg/day) for 24 weeks	47	44.1±9.3	25	30.7±3.5	6	placebo	5
3	Kuchay 2018	25	50.7±12.8	9	30.0±3.8	-	empagliflozin (10 mg/day) for 20 weeks	25	49.1±10.3	8	29.4±3.1	-	placebo	4

Among the three studies included here, two studies report CAP score and LSM^16^, ^17^, three studies report ALT, AST^15^–^17^, while two studies report LDL and TG^15^, ^16^. Jadad scores of the three included studies vary from 4 to 5, and all three studies have high quality according to quality assessment.

### Primary outcomes: CAP score and LSM

**Figure. 2:**
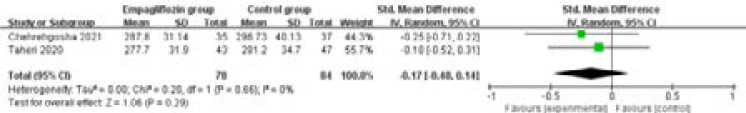
Forest plot for the meta-analysis of CAP score.

**Figure. 3:**
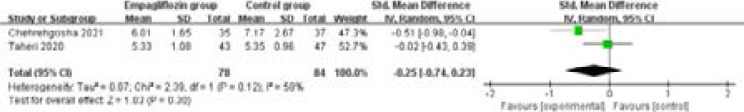
Forest plot for the meta-analysis of LSM score.

These outcome data are analyzed with the random-effects model, and compared to control group for non-alcoholic fatty liver disease, empagliflozin treatment has no obvious effect on CAP score (SMD=-0.17; 95% CI=-0.48 to 0.14; P=0.29) with no heterogeneity among the studies (I2=0%, heterogeneity P=0.29) (Figure 2) or LSM score (SMD=-0.25; 95% CI=-0.74 to 0.23; P=0.30) with significant heterogeneity among the studies (I2=58%, heterogeneity P=0.30) (Figure 3).

### Sensitivity analysis

Significant heterogeneity is observed among the included studies for LSM score, but there are just two RCTs included. Thus, we do not perform sensitivity analysis via omitting one study in turn to detect heterogeneity.

### Secondary outcomes

**Figure. 4:**
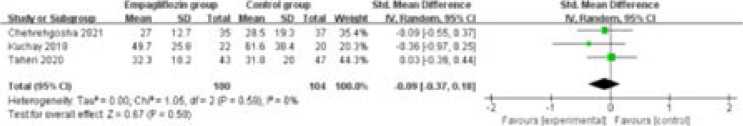
Forest plot for the meta-analysis of ALT.

**Figure. 5:**
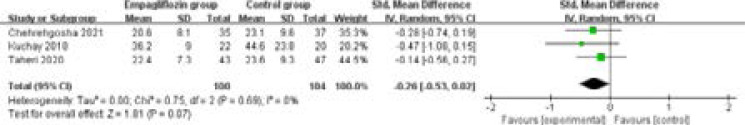
Forest plot for the meta-analysis of AST.

**Figure. 6:**
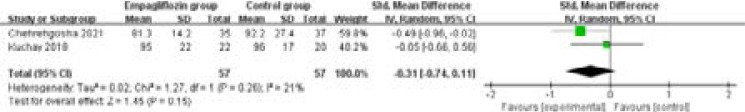
Forest plot for the meta-analysis of LDL.

**Figure. 7:**
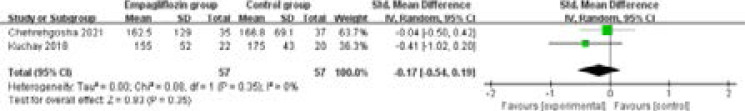
Forest plot for the meta-analysis of TG.

In comparison with control group for non-alcoholic fatty liver disease, empagliflozin treatment shows no substantial impact on ALT (SMD=-0.09; 95% CI=-0.37 to 0.18; P=0.50; Figure 4), AST (SMD=-0.26; 95% CI=-0.53 to 0.02; P=0.07; Figure 5), LDL (SMD=-0.31; 95% CI=-0.74 to 0.11; P=0.15; Figure 6) or TG (SMD=-0.17; 95% CI=-0.54 to 0.19; P=0.35; Figure 7).

## Discussion

Nonalcoholic fatty liver disease commonly occur in patients with type 2 diabetes mellitus which serves as a leading cause of chronic liver disease^22^, ^23^. Type 2 diabetes mellitus is associated with increased risk of cirrhosis, hepatocellular carcinoma, and double the death rate of liver cirrhosis^24^. Liver fat accumulation may result in triglyceride accumulation (steatosis), nonalcoholic steatohepatitis, cirrhosis, and even hepatocellular carcinoma^25^. There are still lack of effective pharmacologic agents for the treatment of nonalcoholic fatty liver disease. Several anti-diabetic agents have been explored considering the importance of insulin resistance for non-alcoholic fatty liver disease, but the results are variable^26^–^28^.

SGLT2 inhibitors are widely used to prevent glucose re-absorption in renal proximal tubules, leading to increased urinary glucose excretion and decreased blood glucose and insulin levels^29^, ^30^. These drugs have the potential in reducing macrovascular events and producing beneficial effects on liver function in both clinical trials and animal models^29^–^32^. They also demonstrate the ability to decrease insulin resistance, adipose tissue dysfunction, and inflammation responses^2^. These provide the theoretical support the benefits of SGLT2 inhibitors to non-alcoholic fatty liver disease. As one important kind of SGLT2 inhibitors, serval studies demonstrated the potential of empagliflozin in treating non-alcoholic fatty liver disease^16^, ^17^.

Our meta-analysis concludes that empagliflozin demonstrates no beneficial effect on hepatic steatosis or fibrosis as shown by the LSM and CAP score in patients with non-alcoholic fatty liver disease. In consistent, no improvements is seen in terms of ALT, AST, LDL or TG after empagliflozin treatment. Regarding the sensitivity analysis, significant heterogeneity remains. Several factors may lead to the heterogeneity. Firstly, the treatment duration of empagliflozin ranges from 20 to 24 weeks. Secondly, two RCTs report patients with diabetes^15^, ^16^, while the remaining RCT report patients without diabetes^17^. Thirdly, there may be some confouning factors such as age, obesity, statin use and blood glucose.

The adverse events of empagliflozin are generally mild and mainly include hypoglycemia, urticaria, fatigue, nocturia and polyuria^15^, ^16^. Our meta-analysis also has some important limitations. Firstly, our analysis is based on three RCTs, and all of them have a relatively small sample size (n<100). Overestimation of the treatment effect was more likely in smaller trials compared with larger samples. There is significant heterogeneity, and empagliflozin treatment may produce variable impact in patients with the comorbidity of diabetes or not. Finally, treatment duration ranges from 20 weeks to 24 weeks, and the duration may be not sufficient to produce the positive results.

## Conclusions

Empagliflozin treatment may provide no additional benefits for non-alcoholic fatty liver disease and should be not recommended in clinical work.
